# Effect of supraphysiological dose of Nandrolone Decanoate on the testis and testosterone concentration in mature and immature male rats: A time course study

**Published:** 2015-12

**Authors:** Rahil Jannatifar, Saeed Shokri, Ahmad Farrokhi, Reza Nejatbakhsh

**Affiliations:** 1 *ACECR Center for Infertility Treatment, Qom Branch, Qom, Iran.*; 2 *Department of Anatomical Sciences, School of Medicine, Zanjan University of Medical Sciences, Zanjan, Iran.*

**Keywords:** *Nandrolone Decanoate*, *Testis*, *Testosterone*, *Mature*, *Immature*, *Rat*

## Abstract

**Background:**

Most studies on anabolic-androgenic steroids abuse have been done in adult rats, but few data are available to immature.

**Objective:**

This study was conducted to assay the effect of Nandrolone Decanoate (ND) on the testis and testosterone concentration in male immature rats compare with mature ones in short and long time.

**Materials and Methods:**

40 mature rats were divided into 4 groups: group A (short term) and group B (long-term) received 10 mg/kg/day ND interaperitoneally for 35 and 70 days, respectively. Group C (control) without any treatment, and group D (vehicle) received dimethyl sulfoxide (DMSO) solution in two periods 35 and 70 days. 40 immature rats were divided into 4 groups same as mature ones. After surgery body weight, testis size, histomorphometry of testis, and serum testosterone level were evaluated.

**Results:**

Our results showed that ND decreased the number of Leydig cells in group B (39.9 ±. 919), group A (43.4 ±. 120), and long term (40.6 ±. 299) immature rats, which could result in a reduction of testosterone concentration significantly in all experimental groups except short term mature group. Number of sertoli cells, testis size, and diameter of seminiferous tubules decreased in the long-term immature group. Eventually, the number of sperm was decreased in mature and immature groups, but a severe depletion of sperm was occurred in both mature and immature in long time in comparison to the control group (p< 0.05).

**Conclusion:**

This time course study showed that supraphysiological dose of ND may negatively affect the number of Leydig cells, sperm cell, and testosterone concentration of immature rats in the same matter of mature rats. However, the number of sertoli cell, testis size, and seminferous diameter were decreased only in the long immature rats.

## Introduction

Androgens play a major role in the development of male reproductive organs such as prostate, penis, seminal vesicle, ductus deferens, and epididymis. This hormone is effective on puberty, fertility, and sexual function in males. 

Leydig cells secret intratesticular testosterone in the testicles. Testosterone is a steroid hormone that has an important role in the development of the male phenotype and the regulation of reproduction of males (1). Physiological levels of this hormone are essential for spermatogenesis (2).

Anabolic-androgenic steroids (AAS) are natural and synthetic compounds that are similar to the male sex hormones. Anabolic-androgenic steroids (ASSs) are used in the treatment of various diseases, such as poor growth, osteoporosis and hypogonadal dysfunction and anemia in mature people (2, 3). Nandrolone decanoate is indicated for the treatment of anemia in immature as well. ASSs in supraphysiological doses have been defined to improve athletic performance (4). However, in recent decades, these compounds have been abused by athletes to increase muscle mass by inducing in protein synthesis (4-7). 

On the other hand, these compounds can lead to reproductive dysfunction and male infertility (4, 8, 9).

AAS abuse is also increasingly common at the younger ages: 2.5% of students 13–14 years old. They have abused AAS, similar to the incidence of crack (2.5%) and heroin (1.6%). The important point is that the response to ASS in adolescents and adults are differently (10).

Almost all body tissues have androgen receptors (AR). Therefore, abuse of AASs may have negative effects on the body systems (11). The side effects of abuse of AASs include liver failure and decrease high density lipoprotein levels, acne vulgaris (11), mood swings, aggressive behavior, and violence (7). The quality of sperm can affect male fertility. Abuse of AAS is a frequent cause of male infertility (12). Some researchers believe that after using AAS, spermatogenesis continues. However, other researchers are believe in ASSs led to a reduction in density, motility and normal morphology of sperm (12) and it would be irreversible.

ASSs are classified in three categories that the first category is Nandrolone Decanoate (ND) (11). ND is chemically synthesized or naturally existed in some vertebrates such as humans (1). High doses of ND interact with various receptors, such as estrogen, progesterone and testosterone. For example, ND-receptor complex can affect gene transcription and increase mRNA and protein in muscle cells (13).

If males treated with ND for a long time, sperm quality and testosterone secretion rate is decreased and the testis shape is altered (1). In fact, the use of intramuscular ND (25 mg/week) suppresses fertility in males (14). The impacts of ND through negative feedback of hypothalamus, lead to testicular atrophy and impaired spermatogenesis and steroidogenesis (9). 

Feinberg *et al* in 1997 showed that high doses of testosterone propionate to peripubertal rats enhanced sexual performance and sexual motivation. However, they could not see the significant effect on sexual behavior of the prepubertal rats. High doses of testosterone propionate decreased the number of Leydig cell in the pre pubertal and adult rats, but had no effect on peripubertal rat (5).

The most AAS users were males in different ages. It is important to determine the effects of suprapgisiological doses of exogenous androgenic hormones on male reproductive organs throughout development. Moreover, few data are available to immature users because most studies on the AAS abuse have been done in adults. The long-term effects of AAS abuse on the immature are even more uncommon. Accordingly, this study was conducted to evaluate the effect of ND on the testis and testosterone concentration in male immature compared with the mature rats in short and long time.

## Materials and methods

**Animals and treatment**

In this experimental study, male rats were divided into mature and immature rats. Mature rats were 8 weeks old and immature rats were 4 weeks old. Forty mature male rats weighing between 200±10 gr and forty immature male rats weighing between 90±5 gr were randomly selected from the laboratory animal center at Qom Azad University. The animals were housed individually in an air-conditioned room (12 hrs. dark/12 hrs. light) at 23 ± 2^º^C and had free access to tap water and standard food pellet. All animal experiments were conducted in accordance with national guidelines and protocols, approved by the Institutional Animal Ethics Committee (IAEC no.03/028/07).

40 mature rats were divided into 4 groups: group A and B, received 10 mg/kg/day ND interaperitoneally (15) for 35 days and 70 days, respectively as short and long term groups. Group C (control group), received normal standard diet, but did not receive any treatment, and group D (vehicle group), received a DMSO solution in two periods, 35 and 70 days. 40 immature rats were divided like mature ones. The ND doses were selected on the basis of reports of nandrolone decanoate use in human (16-18). And, period of administration mimics one cycle of AAS abuse by athletes (11, 19-21). 48 hours after the final drug administration, body weight was measured and the rats were killed rapidly under anesthesia followed by cervical dislocation. Adequate measures were taken to minimize pain and discomfort to the rats and experiments were conducted in accordance with international standards on animal welfare and were also in accordance with local and national regulations.

**Serum testosterone level**

Blood was collected via heart puncture, centrifuged at 2,000 rpm for 10 min and the supernatant (serum) was stored at -20^º^C. The serum was used to measure total serum testosterone, which was determined using AxSYM ® Testosterone test (Abbott, USA). The test is based on Micropartical Enzyme Immunoassay (MEIA) technology for the quantitative determination of total testosterone in serum (22). 

**Weight changes and tissue preparation**

Initial body weight (W_1_) was measured at the beginning of study and final body weight (W_2_) was measured 48 hours after the last injection. The body weight in the experimental groups was measured by body mass= (w_2_-w_1_) /w_1_ × 100.

Then, the rats were scarified and the testis was removed, and fixed at 4% buffered formalin solution. Then, the testes were routinely processed for paraffin embedding; and sections were randomly selected. Samples were placed on the slide and then stained with hematoxylin and eosin (H & E). To count the spermatogenic cells, Leydig cells and Sertoli cells, the method described was used (23). Briefly, In 20 cross sections of seminiferous tubules, the number of nucleoli of cells values per cross section of the seminiferous tubules was quantified, it was possible to calculate the total number of cells per testis using the formula provided by Houchereau-de-Reviers and Lincoln (1978) (24). The calculated value was divided by the testis weight (23). All these observations were performed by optical microscope with bright field (Nikon E800, Japan).

**Sperm count**

Epididymal sperm were collected by dissecting the caudal part of the epididymis. Sperm were separated from epididymal tubules by chopping the caudal part of the epididymis in 5 ml of Hams F10 solution. For reducing bias in different animals, the caudal part of the epididymis was cut from the beginning of the ductus deferens. The solution was incubated for 5 minutes at 37^°^C. The epididymal sperm counts were obtained by the method described in the WHO recommendations (1999). Briefly, the suspension was diluted with saline that contained 0.5% formalin, after which it was placed on an erythrocytometer (Neubauer type) and examined under a light microscope to determine the sperm count.

The data were expressed as total number of sperm/mL (25).

**Statistical analysis**

The data were expressed as the mean values and standard errors (S.E.M). The variables were analyzed by one-way analysis of variance (ANOVA). When a significant effect was found between the groups, Tukey post hoc tests were performed. Testis histomorphometry was analyzed by nonparametric Kruskal-Wallis test followed by Mann-Whitney U test because of non-normally distributed data. 

All analyses were performed using Statistical Package for the Social Sciences, version 16.0, SPSS Inc, Chicago, Illinois, USA (SPSS 16). The statistical significance level was set at p-value≤ 0.05. 

## Results

**Body weight changes**

**Figure 1:**
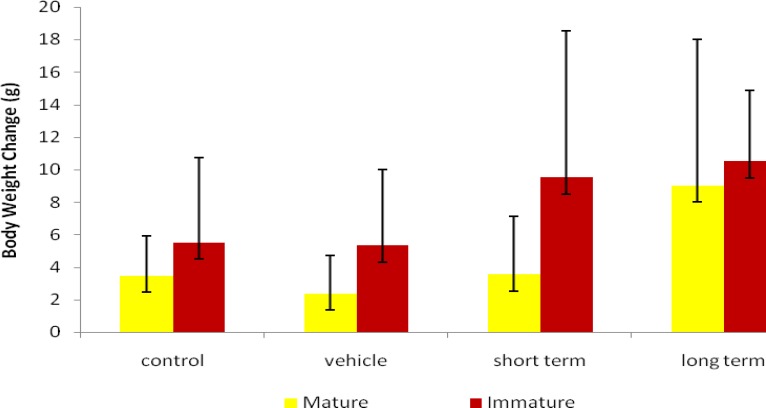
Short and long-term effects of ND on the body weight changes in mature and immature rats

Figure 1 shows that the body weight was increased in all groups during the study. The body weight in long term mature and immature groups and short term immature groups have increased in comparison to the control animals. However, it was not significant in the experimental groups compared to the control group.

**Testis histomorphometry**

The effect of ND on the ratio length to width or size of testis showed a decrease in the long- term immature group in compare with control group (P= 0.04) (Figure 3 and Table I).

Histomorphometry of the testis indicated that the diameter of seminiferous tubule was decreased in long-term immature group (P=0.02) (Table I).

Microscopically alteration in the diameter of tunica albuginea was not observed statistically in all treatment groups (Figure 3 and Table I).

**Testosterone concentration**

**Figure 2:**
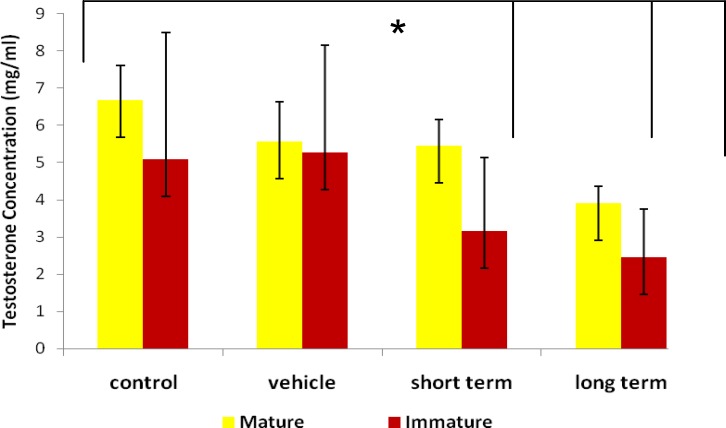
Short and long-term effects of ND on the testosterone concentration in mature and immature rats. * shows that there are significantly differences between control versus short term immature (P=0.04) and long term mature and immature groups (P=0.01) (one-way analysis of variance (ANOVA)).

The level of testosterone showed that ND caused a decrease in the testosterone concentration in long–term mature rats (P=0.04), short and long-term immature rats(P=0.02), in compared with the control and vehicle groups (Figure 2).

**Effects of ND on the Leydig cells, Sertoli cells and spermatogenic cells**

The number of Leydig cells was significantly decreased in all experimental groups except short term mature group in compare with control and vehicle Groups (long term mature: P= 0.03), (short term immature; P= 0.04), (long term immature: P=0.01). (Table II).

Table II shows that the number of Sertoli cells were reduced in long-term immature group compared with control and vehicle groups (P= 0.04). 

Table III shows the number of spermatogonia type A (P= 0.03) and B (P=0.04) was significantly decreased in the long-term mature and immature groups. The number of primary spermatocytes was decreased in all groups (P= 0.02)except in the short-term mature group. The number of spermatids was decreased in long-term mature group, short-term immature group, and long-term immature group (P= 0.01). 

Table III shows that the number of sperm was decreased in mature and immature groups (P= 0.01). But a severe depletion of sperm was occurred in the ND injection for a long time. 

**Table I:** Short and long term effects of Nandrolone Decanoate on the testis in mature and immature rats

**Groups**		**Ratio length to width**			**Diameter of Seminiferous tubules (µ)**				**Diameter of Tunica Albuginea (µ)**			
	**Mature rats ats**	**p-value**	**Immature rats**	**p-value**	**Mature rats**	**p-value**	**Immature rats**	**p-value**	**Mature rats**	**p-value**	**Immature rats**	**p-value**
Control	1.39± 0.056		1.37 ± 0.063		62.2 ± 2.619		1.37 ± 0.063		62.2 ± 2.619		1.37 ± 0.063	
Vehicle	1.2 ± 0.056		1.271 ± 0.066		62 ± 2.952		1.271 ± 0.066		62 ± 2.952		1.271 ± 0.066	
Short term	1.33 ± 0.058	0.15	1.3 ± 0.063	0.2	59.6 ±1.9	0.15	1.3 ± 0.063	0.2	59.6 ±1.9	0.15	1.3 ± 0.063	0.2
Long term	1.26 ± 0.052	0.09	1.092 ± 0.062^a^	0.04	53.4 ±2.234^b^	0.09	1.092 ± 0.062^a^	0.04	53.4 ±2.234^b^	0.09	1.092 ± 0.062^a^	0.04

All data are presented as Mean ± SEM. P-values show statistically differences between all experimental groups versus control (one-way analysis of variance (ANOVA).)

**Table II:** Short and long term effects of Nandrolone Decanoate on the number of Leydig cells and Sertoli cells in mature and immature rats

**Groups**	**Number of Leydig cell**				**Number of Sertoli cell**			
	**Mature rats**	**p-value**	**Immature rats**	**p-value**	**Mature rats**	**p-value**	**Immature rats**	**p-value**
Control	47.9± 1.132		47.15± 0.921		2.5± 0.211			
Vehicle	47.7± 1.138		39.9± 1.919^a^		47.7± 1.138		3.2 ± 0.277	
Short-term	47.15± 0.921	0.22	47.9± 1.132	47.15± 0.921	47.15± 0.921	0.12	2.9 ± 0.260	0.22
Long –term	39.9± 1.919^a^	0.03	47.7± 1.138	47.15± 0.921	47.9± 1.132	0.9	2.05± 0.198^d^	0.03

All data are presented as Mean ± SEM. P-values show statistically differences between all experimental groups versus control (one-way analysis of variance (ANOVA).).

**Table III:** Short and long term effects of Nandrolone Decanoate on the number of spermatogenic cells (spermatogonia A, spermatogonia B, primary spermatocyte, spermatid, and sperm) in mature and immature rats

**Group**	**Number of spermatogonia type A**		**Number of spermatogonia type B**		**Number of primary spermatocyte**		**Number of spermatid**		**Number of sperm**	
	M	I	M	I	M	I	M	I	M	I
Control	2.85±0.26	3.1±0.30	3.1±0.25	3.1±0.30	3.1±0.30	3.1±0.25	2.85±0.26	3.1±0.30	3.1±0.30	3.1±0.30
Vehicle	2.65±0.22	3±0.29	3±0.29	3±0.29	3±0.29	3±0.29	2.65±0.22	3±0.29	3±0.29	3±0.29
Short-term	2.6±0.21	2.8±0.26	2.8±0.26	2.8±0.26	2.8±0.26	2.8±0.26	2.6±0.21	2.8±0.26	2.8±0.26	2.8±0.26
Long –term	1.9±0.16^a^	2.05±0.18^a^	2.1±0.19^b^	2.05±0.18^a^	2.05±0.18^a^	2.1±0.19^b^	1.9±0.16^a^	2.05±0.18^a^	2.05±0.18^a^	2.05±0.18^a^

All data are presented as …….. a (p-value=0.03), b (p= 0.04), c (p= 0.02), d (p= 0.01), and e (p=0.01) show that there are significantly differences between those experimental rats versus control group. The other experimental groups are not statistically significant (one-way analysis of variance (ANOVA).)). M: Mature rats, I: Immature rats.

**Figure 3:**
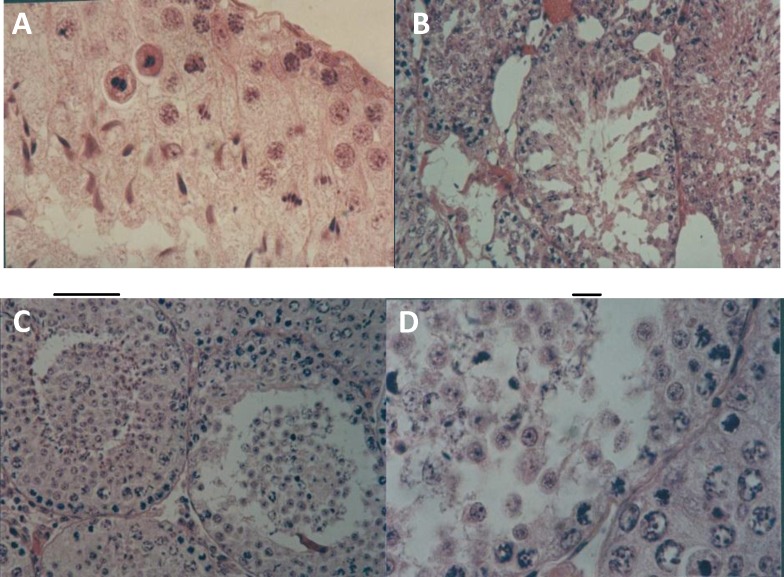
Seminiferous tubule of A, Control (× 100), B; long term immature (× 40), C and D, long term mature that disorder in spermatogenetic cells occurred (× 40, ×100)

## Discussion

To strengthen the anabolic properties of testosterone, more than 100 synthetic steroid derivatives have been described for human purposes, anabolic effect promotes protein synthesis, muscle growth and erythropoietin (9). The present study elucidated spermatogenesis aspects of ND as an androgen derivative in high doses on mature and immature rats for long and short time.

This study showed the body weight in long term mature and immature groups and short term immature groups have increased in compared with the control ones although it wasn’t statistically significant. Others reported that an increase in body weight may be ascribed to the reposition of fluids and sodium in the body (26). The few investigations showed that the body weight gain is indirectly the result of decreasing fatigue and increasing anxiety, which may increase the exercise tolerance by skeletal muscles at the end days ND using for a long time. Therefore, body fat was reduced or was probably the result of a lowered food intake (27, 28).

In this study, the ratio of length to width or size of the testis of experimental long term mature and immature groups were significantly lower than those in the control and vehicle groups. Estimation of testicular volume is a good indicator of testicular atrophy. The decrease in testis size might be a consequence of reduction in seminiferous tubule length (29). The use of high doses of nandrolone reduced testicular size and length of seminiferous tubules in the rats (30).

Like other tissues, Androgens act in the testis by activating androgen receptor (AR) transcription (13). Inside Sertoli cells, testosterone binds to ARs at the beginning of its activation, cause activation of the receptor to maintains spermatogenesis and inhibits apoptosis of germ cells (2).

AR expression is maintained by endogenous testicular androgens, absence of testosterone is known to lead to disruption of spermatogenesis (11). ND injected with low or high doses, can lead to reduce of testosterone, which caused the maturation arrest at the primary spermatocyte level and spermatid level (11). 

It is clearly known that, gonadotropin releasing hormones from the pituitary gland (LH, FSH) have growth promoting effects on testis development. Therefore, a decrease in LH release from the pituitary gland, may in turn result in a decreased testosterone level, and as a result, testicular atrophy occurs (31). In this regard, the present study, showed that the size of the testis of long-term mature and immature groups were lower than those of in the control group. Suprophysiological doses of ND were caused interaction between ND and AR in cells (13). ND-receptor complex affected hypothalamus- pituitary-gonadal axis by negative feedback and reduced the level of LH and FSH. 

A decrease of LH form pituitary gland showed that Leydig cells were reduced and receptors on Leydig cell that interaction with LH was inactive. Following production of testosterone was decreased, which closely resemble with present study that the level of testosterone was decreased in the long- term mature and immature groups. Because of decreasing the level of FSH, Sertoli cells had not sufficient growth and development (32, 33). Therefore, our study declared depletion in the number of them in long term immature groups.

In Sertoli cells, testosterone selectively binds to the ARs. activation of the receptor maintain spermatogenesis and inhibit germ cell apoptosis (11). In the testis, ARs are expressed in the somatic leydig cells, per tubular myoid cells and Sertoli cells as well as to rete testis, the epithelial cells of the epididymis, and prostate (32, 33). Sertoli cells play an important role in somatic cell organization and in determining the structure of the testis (11). 

They also supported a large number of germ cells, and thus their numbers in adult determined spermatogenic capacity. The present results showed that AAS caused reduction in the number of Sertoli cells, which might be due to the structural response of Sertoli cells to testosterone deprivation (11). 

Type A and B of Spermatogonia were increased by mitosis division. Increasing of these cells depended on supporting of Sertoli cells. When sertoli cells were inactive they were not stimulated to regulate the division and maintained of spermatogenic cells. Kerr reported that suppression of spermatogenesis is apparently caused by suppression of pituitary gonadotropin secretion (34) whereas the present results showed spermatogonia type A and B are lacking in the number of long-term mature group and all immature groups. 

Following decrease of Spermatogonia type A and B, the number of primary spermatocytes and spermatids were reduced in the same groups. The number of sperm by treatment ND were decreased in all groups, but the severe reduction was occurred for long time injection. 

Results of other studies revealed that sperm parameters such as sperm count, motility and normal morphology deteriorated in groups that have received ND (8).

In fact, in rats that had been received ND compared with the control and sham groups, testis weight and reproductive organs significantly were decreased. Moreover, fertility parameters such as sperm count, motility and normal morphology compare with control and vehicle groups reduced (35). Karbalay-Doust *et al.* in 2007 showed that the use of nandrolone in rats caused structural changes in the testis, sperm quality, and the length of seminiferous tubules and reduced the weight of the prostate gland (36).

## Conclusion

In conclusion, This time course study showed that supraphysiological doses of Nandrolone Decanoate may negatively affect the number of Leydig cells, sperm cell and testosterone concentration of immature rats in the same matter of mature rats. However, the number of sertoli cell, testis size and seminferous diameter were decreased only in the long immature rats.
